# The effects of chemotherapy, primary tumor location and histological subtype on the survival of stage III colon cancer patients

**DOI:** 10.1186/s12876-023-02741-3

**Published:** 2023-04-05

**Authors:** Chenghui Zhou, Liqing Lu, Qiulin Huang, Zhen Tang, Rong Tang, Zhongsheng Xiao, Shuai Xiao

**Affiliations:** 1 Department of General Surgery, Xiangya Hospital Central South University, Central South University, Changsha, China; 2 Department of General, Visceral, Cancer and Transplantation Surgery, University Hospital Cologne, Cologne, Germany; 3 Key Laboratory of Cancer Proteomics of Chinese Ministry of Health, Xiangya Hospital, Central South University, Changsha, 410008 Hunan China; 4 The First Affiliated Hospital, Department of Gastrointestinal Surgery, Hengyang Medical School, University of South China, Hengyang, China; 5 The First Affiliated Hospital, Institute of Oncology, Hengyang Medical School, University of South China, Hengyang, China

**Keywords:** Colon cancer, Chemotherapy, Primary tumor location, Mucinous adenocarcinoma, Overall survival

## Abstract

**Objective:**

Colon cancer (CC) is one of the most common cancers worldwide and has a poor prognosis. Surgery followed by adjuvant chemotherapy is the standard treatment strategy for stage III CC patients. Primary tumor location (PTL) is an important factor for the long-term survival of CC. However, the difference in the prognosis between the histological subtypes of mucinous adenocarcinoma (MAC) and nonspecific adenocarcinoma (AC) in stage III CC patients is unclear. The correlation of chemotherapy, PTL and histological subtype with the overall survival (OS) of stage III CC patients has not yet been explored.

**Methods:**

Patients diagnosed with stage III CC from 2010 to 2016 in the Surveillance, Epidemiology, and End Results (SEER) database were retrieved. The clinicopathological features and OS were analyzed according to the chemotherapy, PTL and histological subtype.

**Results:**

A total of 28,765 eligible stage III CC patients were enrolled in this study. The results showed that chemotherapy, left-sided CC (LCC) and AC were favorable prognostic factors for OS. Right-sided CC (RCC) had worse OS than LCC regardless of chemotherapy. MAC had worse OS than AC in the patients with chemotherapy, but the survival benefits disappeared in the patients without chemotherapy. Additionally, in LCC, MAC had worse OS than AC regardless of chemotherapy. However, in RCC, MAC had worse OS than AC in patients with chemotherapy but had similar OS to AC in patients without chemotherapy. In the AC group, RCC had worse OS than LCC regardless of chemotherapy. In the MAC group, RCC had comparable OS to LCC regardless of chemotherapy. Four subgroups, i.e., RCC/MAC, RCC/AC, LCC/MAC and LCC/AC, all showed benefits from chemotherapy. Among them, LCC/AC had the best OS, and RCC/MAC had the worst OS compared with the other three subgroups.

**Conclusion:**

The prognosis of MAC is worse than that of AC in stage III CC. LCC/AC has the best OS, while RCC/MAC has the worst OS but still benefits from chemotherapy. The impact of chemotherapy on survival is greater than that of histological subtype, but the impact of histological subtype on survival is similar to that of PTL.

## Background

Colon cancer (CC) remains one of the most common and deadly cancers worldwide [1, 2]. In particular, the majority of CC patients are diagnosed at an advanced stage, especially stage III. Adjuvant chemotherapy following surgery is the recommended treatment for stage III CC patients [3, 4]. Previous studies have reported that primary tumor location (PTL) could significantly affect the efficacy of chemotherapy and the long-term survival of CC patients, with worse survival in right-sided CC (RCC) patients than in left-sided CC (LCC) patients [5, 6]. Additionally, evidence has also shown that RCC has impaired sensitivity to chemotherapy compared with LCC [7, 8]. Studies have suggested that potential factors such as microsatellite instability-high (MSI-H) and BRAF mutation status in PTL lead to such differences, but the exact molecular mechanisms have yet to be fully elucidated [9, 10].

Recently, the specific histological subtype of mucinous adenocarcinoma (MAC) of CC has become a hot research topic [11, 12]. MAC is the second most common histological subtype and accounts for approximately 10–15% of all CC patients, which is defined as more than 50% of tumor volume being composed of extracellular mucin [13, 14]. MAC has some distinct clinicopathological features, such as more RCC, advanced tumor stage, and frequent MSI-H, CpG island methylator phenotype (CIMP) and BRAF mutation [15]. Interestingly, a previous study demonstrated that MAC is associated with chemo-resistance compared with nonspecific adenocarcinoma (AC), especially for 5-FU-based chemotherapy regimens [16]. Therefore, it has become a new topic in current research on chemotherapy for CC.

Chemotherapy resistance usually occurs in RCC [17]. Accordingly, MAC is always common in RCC and presents a chemoresistance tendency [18, 19]. More interestingly, some studies have shown that RCC and MAC have some similar molecular characteristics [20, 21]. Therefore, PTL and histological subtype are two unmodifiable factors associated with patient survival who receive chemotherapy, which has received increasing attention [10]. All the above interesting findings led us to explore the cross-linked impact of PTL and histological subtype on the survival of stage III CC patients who were treated with radical surgery plus adjuvant chemotherapy. Therefore, this study aimed to explore the main risk factors affecting the survival of stage III CC patients in a cross-linked condition of chemotherapy, PTL and histological subtype using the Surveillance, Epidemiology, and End Results (SEER) database.

## Materials and methods

### Data source

As we described previously, we required cases from 18 SEER registries (http://seer.cancer.gov/csr/1975_2017) and obtained permission to download the data from the SEER database, which did not require informed patient consent [22].

### Patient selection

**Fig. 1:**
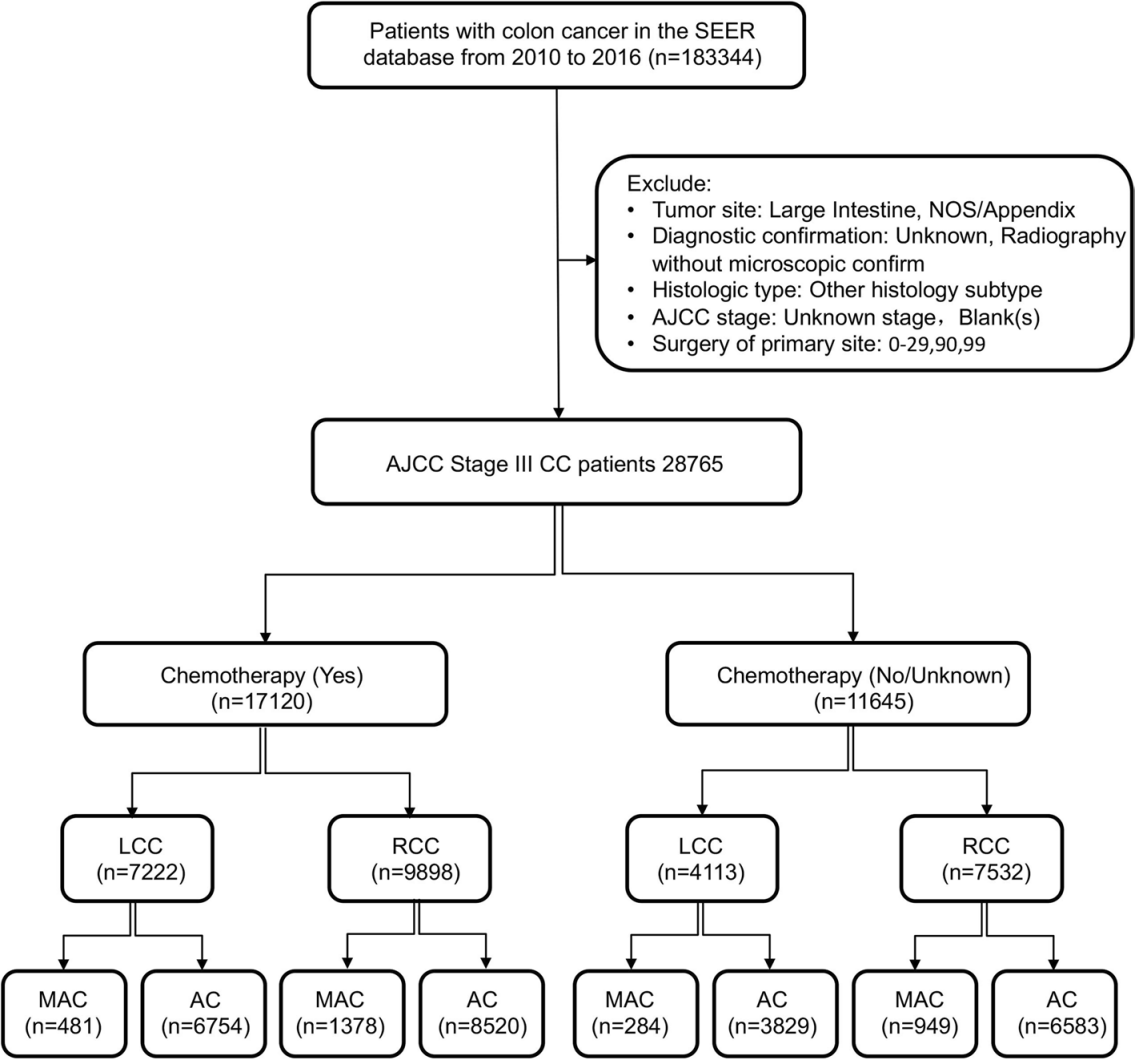
Flowchart of patient inclusion and exclusion into the study. SEER, Surveillance, Epidemiology, and End Results; AJCC, American Joint Committee on Cancer; RCC, right-sided colon cancer; LCC, left-sided colon cancer; AC, nonspecific adenocarcinoma; MAC, mucinous adenocarcinoma

We accessed the SEER database (SEER*Stat 8.3.6) and the patients diagnosed with III colon cancer from 2010 to 2016 were enrolled (Fig. 1). This study included colon cancer patients: 1) pathological evidence was confirmed; 2) complete clinicopathological information was available in the database; 3) American Joint Committee on Cancer (AJCC) 7^th^ ed stage = III; and 4) the primary tumor sites were from cecum to sigmoid colon. Patients were excluded if 1) the tumor site was large intestine and NOS/Appendix; 2) diagnostic confirmation was unknown and radiography without microscopic confirmation; 3) the histologic type was another histological subtype; 4) the AJCC stage was unknown and blank(s); and 5) surgery at the primary site was 0–29, 90 and 99.

### Statistical analysis

Statistical analyses were performed with SPSS (SPSS version 22.0, USA). The clinicopathological characteristics of stage III CC patients were compared using the chi-square test. OS of CC patients was estimated using Kaplan–Meier survival curves, and the log-rank test was used among these groups. The multivariate Cox regression analyses were employed to identify factors effected stage III CC patients’ OS. *P* value of less than 0.05 was considered statistically significant.

## Results

### General characteristics of patients with AJCC stage III CC

A total of 28,765 eligible cases were retrieved and enrolled from the SEER database according to the inclusion and exclusion criteria. All common clinicopathologic features between RCC and LCC and between AC and MAC are shown in Table 1 and Table 2, respectively. A total of 60.6% of patients (*n* = 17,430) were RCC patients, 39.4% (*n* = 11,335) were LCC patients, 89.3% (*n* = 25,686) were AC patients, and 10.7% (*n* = 3079) were MAC patients. The differences in demographic and clinicopathological characteristics between the RCC group and the LCC group were statistically significant in terms of sex, age, histological subtype, grade, p–T/N stage, chemotherapy, tumor size, tumor deposits and perineural invasion, as shown in Table 1 (*P* ≤ *0.001*). Among those patients, RCC patients were more likely to be diagnosed with MAC than LCC patients (*P* ≤ *0.001*).

**Table 1:** Characteristics of patients with AJCC stage III colon cancer by PTL

	Total(28,765)	RCC (60.6%)	LCC (39.4%)	*P*/Value
**Sex**				≤ 0.001
Male	13,994 (48.6)	7903 (45.3)	6091 (53.7)	
Female	14,771 (51.4)	9527 (54.7)	5244 (46.3)	
**Age**				≤ 0.001
≤ 60	8791 (30.6)	4097 (23.5)	4694 (41.4)	
> 60	19,974 (69.4)	13,333 (76.5)	6641 (58.6)	
**Histological subtype**				≤ 0.001
AC	25,686 (89.3)	15,103 (86.6)	10,583 (93.4)	
MAC	3079 (10.7)	2327 (13.4)	752 (6.6)	
**Grade**				≤ 0.001
Grade I/II	20,696(71.9)	11,578(66.4)	9118(80.4)	
Grade III/IV	7619(26.5)	5575 (32.0)	2044 (18.0)	
Unknown	450(1.6)	277 (1.6)	173 (1.5)	
**p–T Stage**				≤ 0.001
T1	614(2.1)	310(1.8)	304(2.7)	
T2	2099(7.3)	1202(6.9)	897(7.9)	
T3	19,125(66.5)	11,507(66.0)	7618 (67.2)	
T4	6886(23.9)	4387(25.2)	2499(22.0)	
TX	41(0.1)	24(0.1)	17(0.1)	
**p-N Stage**				≤ 0.001
N1	19,095(66.4)	11,350(48.6)	7745(68.3)	
N2	9670(33.6)	6080(34.9)	3590(31.7)	
**Chemotherapy**				≤ 0.001
No/Unknown	11,645(40.5)	7532(43.2)	4113(36.3)	
Yes	17,120(59.5)	9898(56.8)	7222(63.7)	
**Tumor size**				≤ 0.001
≤ 5	17,329(60.2)	9718(55.8)	7611(67.1)	
> 5	10,826(37.6)	7391(42.4)	3435(30.3)	
Unknown	610(2.1)	321(1.8)	289(2.5)	
**CEA**				0.525
Normal	9756(33.9)	5871(33.7)	3885(34.3)	
Elevated	7753(27.0)	4699(27.0)	3054(26.9)	
Borderline/Unknown	11,256(39.1)	6860(39.4)	4396(38.8)	
**Tumor deposit**				≤ 0.001
None	20,965(72.9)	12,926(74.2)	8039(70.9)	
Yes	6147(21.4)	3535(20.3)	2612(23.0)	
Unknown	1653(5.7)	969(5.6)	684(6.0)	
**Perineural invasion**				≤ 0.001
None	21,560(75.0)	13,278(76.2)	8282(73.1)	
Yes	4657(16.2)	2635(15.1)	2022(17.8)	
Unknown	2548(8.9)	1517(8.7)	1031(9.1)

**Table 2:** Characteristics of patients with AJCC stage III colon cancer by histological subtype

	Total(28,765)	AC (89.3%)	MAC (10.7%)	P/Value
**Sex**				0.166
Male	13,994 (51.4)	12,522 (48.8)	1472 (47.8)	
Female	14,771 (48.6)	13,164 (51.2)	1607 (52.2)	
**Age**				0.015
≤ 60	8791 (30.6)	7909 (30.8)	882 (28.6)	
> 60	19,974 (69.4)	17,777 (69.2)	2197 (71.4)	
**PTL**				≤ 0.001
RCC	17,430 (60.6)	15,103 (58.8)	2327 (75.6)	
LCC	11,335 (39.4)	10,583 (41.2)	752 (24.4)	
**Grade**				≤ 0.001
Grade I/II	20,696 (71.9)	18,657 (72.6)	2039 (66.2)	
Grade III/IV	7619 (26.5)	6713 (26.1)	906 (29.4)	
Unknown	450 (1.6)	316 (1.2)	134 (4.4)	
**p–T Stage**				≤ 0.001
T1	614 (2.1)	558 (2.2)	56 (1.8)	
T2	2099 (7.3)	1916 (7.5)	183 (5.9)	
T3	19,125 (66.5)	17,206 (67.0)	1919 (62.3)	
T4	6886 (23.9)	5970 (23.2)	916 (29.7)	
TX	41 (0.1)	36 (0.1)	5 (0.1)	
**p-N Stage**				≤ 0.001
N1	19,095 (66.4)	17,199 (67.0)	1896 (61.6)	
N2	9670 (33.6)	8487 (33.0)	1183 (38.4)	
**Chemotherapy**				0.307
No/Unknown	11,645 (40.5)	10,412 (40.5)	1233 (40.0)	
Yes	17,120 (59.5)	15,274 (59.5)	1846 (60.0)	
**Tumor size**				≤ 0.001
≤ 5	17,329 (60.2)	16,018 (62.4)	1311 (42.6)	
> 5	10,826 (37.6)	9113 (35.5)	1713 (55.6)	
Unknown	610 (2.1)	555 (2.2)	55 (1.8)	
**CEA**				0.017
Normal	9756 (33.9)	8780 (34.2)	976 (31.7)	
Elevated	7753 (27.0)	6880 (26.8)	873 (28.4)	
Borderline/Unknown	11,256 (39.1)	10,026 (39.0)	1230 (39.9)	
**Tumor deposit**				0.823
None	20,965 (72.9)	18,708 (72.8)	2257 (73.3)	
Yes	6147 (21.4)	5496 (21.4)	651 (21.2)	
Unknown	1653 (5.7)	1482 (5.8)	171 (5.6)	
**Perineural invasion**				≤ 0.001
None	21,560 (75.0)	19,103 (74.4)	2457 (79.8)	
Yes	4303 (16.8)	4303 (16.8)	354 (11.5)	
Unknown	2548 (8.9)	2280 (8.9)	268 (8.7)	

As shown in Table 2, the differences in demographic and clinicopathological characteristics between the MAC group and the AC group were statistically significant in terms of age, PTL, grade, p–T stage, tumor size and perineural invasion (*P* < 0.05). Patients with MAC were more likely to be > 60 years old, RCC, grade III/IV, T4 tumors, tumor size > 5 cm, elevated CEA, and less perineural invasion than patients with AC.

### Long-term survival of patients with AJCC stage III colon cancer according to chemotherapy, PTL and histological subtype

**Fig. 2:**
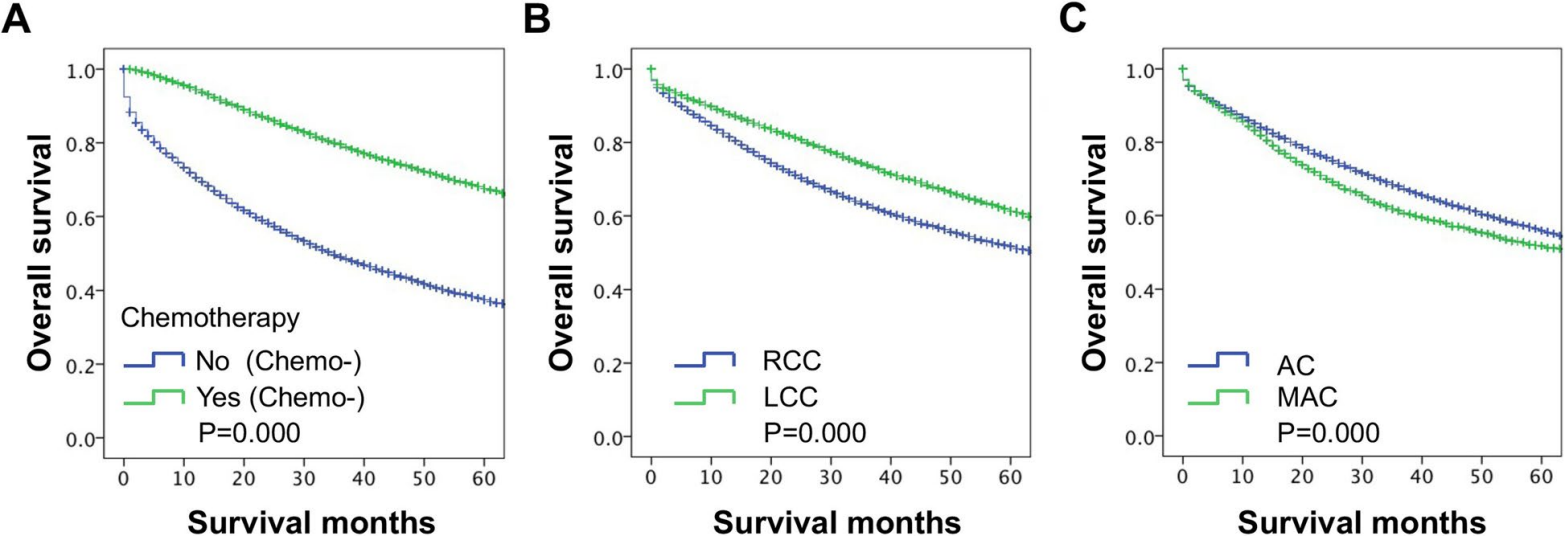
Long-term survival of patients with stage III CC according to chemotherapy, PTL and histology. **A** The survival curves showed that the OS of the nonchemotherapy patients was poorer than that of the chemotherapy patients. **B** The survival curves showed that the OS of RCC patients was poorer than that of LCC patients. **C** The survival curves showed that the OS of MAC patients was poorer than that of AC patients

To explore the potential advantage of PTL, histological subtype and chemotherapy for long-term survival, we analyzed the survival difference via Kaplan‒Meier analysis and log-rank tests. The results showed that the OS of the nonchemotherapy group was poorer than that of the chemotherapy group (*P* ≤ 0.001, Fig. 2A); the OS of the RCC group was poorer than that of the LCC group (*P* ≤ *0.001*, Fig. 2B), and the OS of the MAC group was poorer than that of the AC group (*P* ≤ *0.001*, Fig. 2C). These results indicated that subgroups such as chemotherapy, LCC and AC were favorable prognostic factors affecting the OS of patients with stage III CC.

### The impact of PTL and histological subtype on the survival of patients with stage III CC stratified by chemotherapy.

**Fig. 3:**
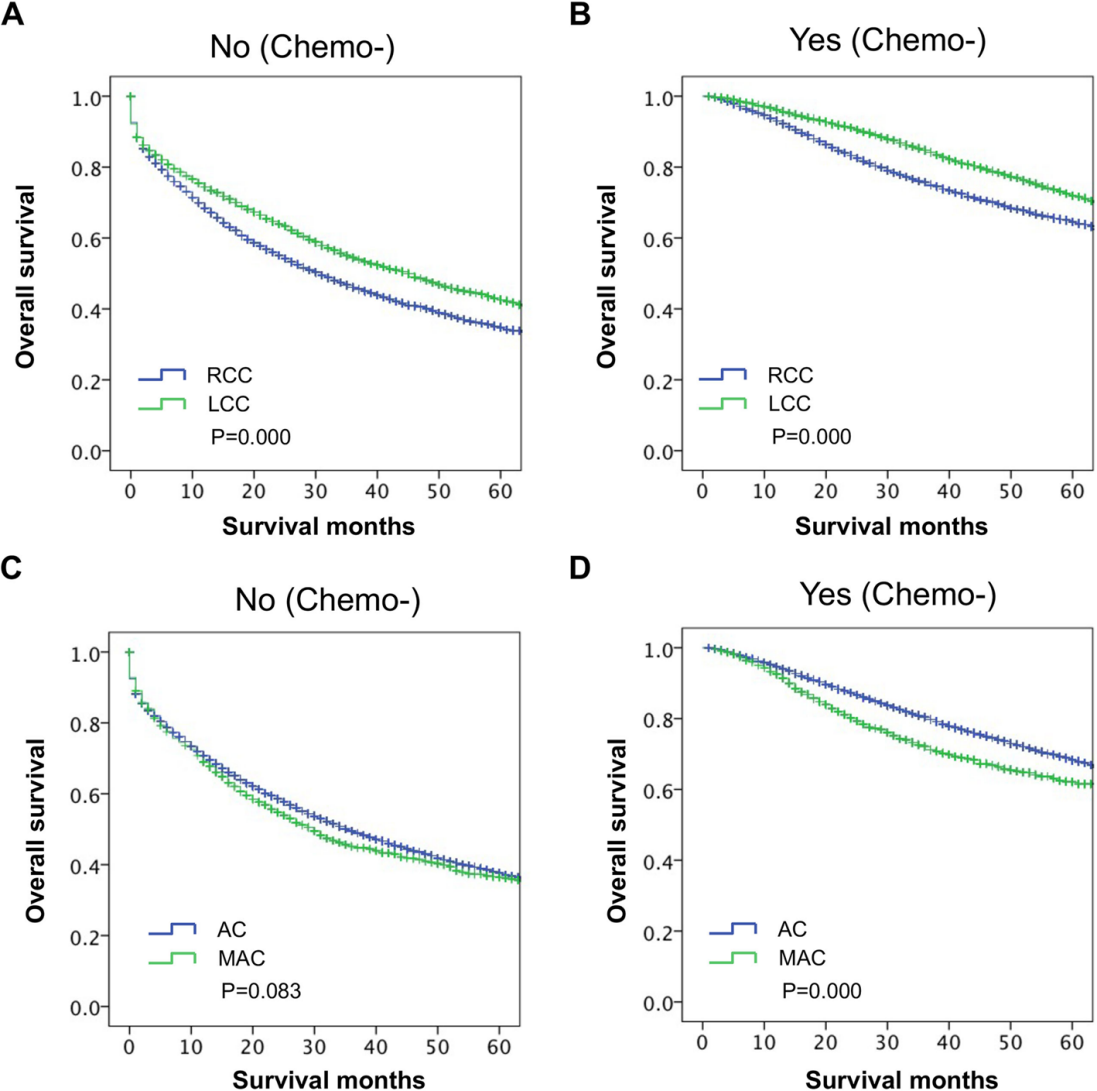
The impact of PTL and histological subtype on the survival of patients with stage III CC stratified by chemotherapy. **A** For nonchemotherapy patients, LCC had better OS than RCC. **B** For chemotherapy patients, LCC had better OS than RCC. **C** For nonchemotherapy patients, AC patients had comparable OS with MAC patients. **D** For chemotherapy patients, AC patients had better OS than MAC patients with chemotherapy

We previously found that patients with stage III CC who received chemotherapy had better OS than those who did not receive chemotherapy. Additionally, the OS of the RCC group was lower than that of the LCC group. We further analyzed the survival differences between different PTL via stratification of chemotherapy treatment. The distribution of chemotherapy among them is shown in Table 3. The results showed that the OS of the RCC group was worse than that of the LCC group in the patients without chemotherapy (*P* ≤ *0.001*, Fig. 3A). Furthermore, the OS of the RCC group was also worse than that of the LCC group in the patients with chemotherapy (*P* ≤ *0.001*, Fig. 3B). These results indicated that PTL might be an independent prognostic factor affecting the OS of patients with stage III CC regardless of chemotherapy.

**Table 3:** Characteristics AJCC stage III CC patients with chemotherapy treatment stratified by PTL

	Total (17,120)	Chemotherapy		*P*/Value
		RCC (9898)	LCC (7222)	
**Sex**				0.166
Male	8598 (50.2)	4702 (47.5)	3896 (53.9)	
Female	8522 (49.8)	5196 (52.5)	3326 (53.9)	
**Age**				≤ 0.001
≤ 60	7018 (41.0)	3261 (32.9)	3757 (52.0)	
> 60	10,102 (59.0)	6637 (67.1)	3465 (48.0)	
**Histological subtype**				≤ 0.001
AC	15,274 (89.2)	8520 (86.1)	6754 (93.5)	
MAC	1846 (10.8)	1378 (13.9)	468 (6.5)	
**Grade**				≤ 0.001
Grade I/II	12,502 (73.0)	6685 (67.5)	5817 (80.5)	
Grade III/IV	4356 (25.4)	3059 (30.9)	1297 (18.0)	
Unknown	262 (1.5)	154 (1.6)	108 (1.5)	
**p–T Stage**				≤ 0.001
T1	391 (2.3)	180 (1.8)	211 (2.9)	
T2	1292 (7.5)	686 (6.9)	606 (8.4)	
T3	11,442 (66.8)	6627 (67.0)	4815 (66.7)	
T4	3967 (23.2)	2390 (24.1)	1577 (21.8)	
TX	28 (0.2)	15 (0.2)	13 (0.2)	
**p-N Stage**				≤ 0.001
N1	11,112 (64.9)	6283 (63.5)	4829 (66.9)	
N2	6008 (35.1)	3615 (36.5)	2393 (33.1)	
**Tumor size**				≤ 0.001
≤ 5	10,567 (61.7)	5690 (57.5)	4877 (67.5)	
> 5	6171 (36.0)	4022 (40.6)	2149 (29.8)	
Unknown	382 (2.2)	186 (1.9)	196 (2.7)	
**CEA**				0.893
Normal	6595 (38.5)	3798 (38.4)	2797 (38.7)	
Elevated	4813 (28.1)	2790 (28.2)	2023 (28.0)	
Borderline/Unknown	5712 (33.4)	3310 (33.4)	2402 (33.3)	
**Tumor deposit**				≤ 0.001
None	12,577 (73.5)	7392 (74.7)	5185 (71.8)	
Yes	3661 (21.4)	1999 (20.2)	1662 (23.0)	
Unknown	882 (5.2)	507 (5.1)	375 (5.2)	
**Perineural invasion**				≤ 0.001
None	12,946 (75.6)	7661 (77.4)	5285 (73.2)	
Yes	2880 (16.8)	1513 (15.3)	1367 (18.9)	
Unknown	1294 (7.6)	724 (7.3)	570 (7.9)

The OS of the MAC group was lower than that of the AC group in our study. Then, we further analyzed the survival differences between AC and MAC patients via stratification of chemotherapy. The distribution of chemotherapy among them is shown in Table 4. The results showed that the OS of the MAC group was similar to that of the AC group in patients without chemotherapy (*P* = 0.083, Fig. 3C). However, the OS of the MAC group was worse than that of the AC group in the patients who received chemotherapy (*P* ≤ *0.001*, Fig. 3D). These results indicated that in the chemotherapy subgroup, histological subtype might be a prognostic factor for patients with stage III CC.

**Table 4:** Characteristics AJCC stage III CC patients with chemotherapy treatment stratified by histological subtype

	Total (17,120)	Chemotherapy		*P*/Value
		AC (9898)	MAC (7222)	
**Sex**				0.251
Male	8598 (50.2)	7685 (50.3)	913 (49.5)	
Female	8522 (49.8)	7589 (49.7)	933 (50.5)	
**Age**				0.026
≤ 60	7018 (41.0)	6306 (41.3)	712 (38.6)	
> 60	10,102 (59.0)	8968 (58.7)	1134 (61.4)	
**PTL**				≤ 0.001
RCC	9898 (57.8)	8520 (55.8)	1378 (74.6)	
LCC	7222 (42.2)	6754 (44.2)	468 (25.4)	
**Grade**				≤ 0.001
Grade I/II	12,502 (73.0)	11,266(73.8)	1236(67.0)	
Grade III/IV	4356 (25.4)	3819 (25.0)	537 (29.1)	
Unknown	262 (1.5)	73 (4.0)	189 (1.2)	
**p–T Stage**				≤ 0.001
T1	391 (2.3)	360 (2.4)	31 (1.7)	
T2	1292 (7.5)	1179 (7.7)	113 (6.1)	
T3	11,442 (66.8)	10,296 (67.4)	1146 (62.1)	
T4	3967 (23.2)	3415 (22.4)	552 (29.9)	
TX	28 (0.2)	24 (0.2)	4 (0.2)	
**p-N Stage**				≤ 0.001
N1	11,112 (64.9)	10,000 (65.5)	1112 (60.2)	
N2	6008 (35.1)	5274 (34.5)	734 (39.8)	
**Tumor size**				≤ 0.001
≤ 5	10,567 (61.7)	9757 (63.9)	810 (43.9)	
> 5	6171 (36.0)	5166 (33.8)	1005 (54.4)	
Unknown	382 (2.2)	351 (2.3)	31 (1.7)	
**CEA**				0.088
Normal	6595 (38.5)	5927 (38.8)	668 (36.2)	
Elevated	4813 (28.1)	4279 (28.0)	534 (28.9)	
Borderline/Unknown	5712 (33.4)	5068 (33.2)	644 (34.9)	
**Tumor deposit**				0.265
None	12,577 (73.5)	11,217 (73.4)	1360 (73.7)	
Yes	3661 (21.4)	3256 (21.3)	405 (21.9)	
Unknown	882 (5.2)	801 (5.2)	81 (4.4)	
**Perineural invasion**				≤ 0.001
None	12,946 (75.6)	11,461 (75.0)	1485 (80.4)	
Yes	2880 (16.8)	2642 (17.3)	238 (12.9)	

### The impact of the correlation between PTL and histological subtype on the survival of stage III CC patients with chemotherapy.

PTL and histological subtype were identified as prognostic factors for stage III CC patients with chemotherapy in our study. Then, we further analyzed the survival differences between PTL and histological subtype via stratification analyses in the chemotherapy group.

**Fig. 4:**
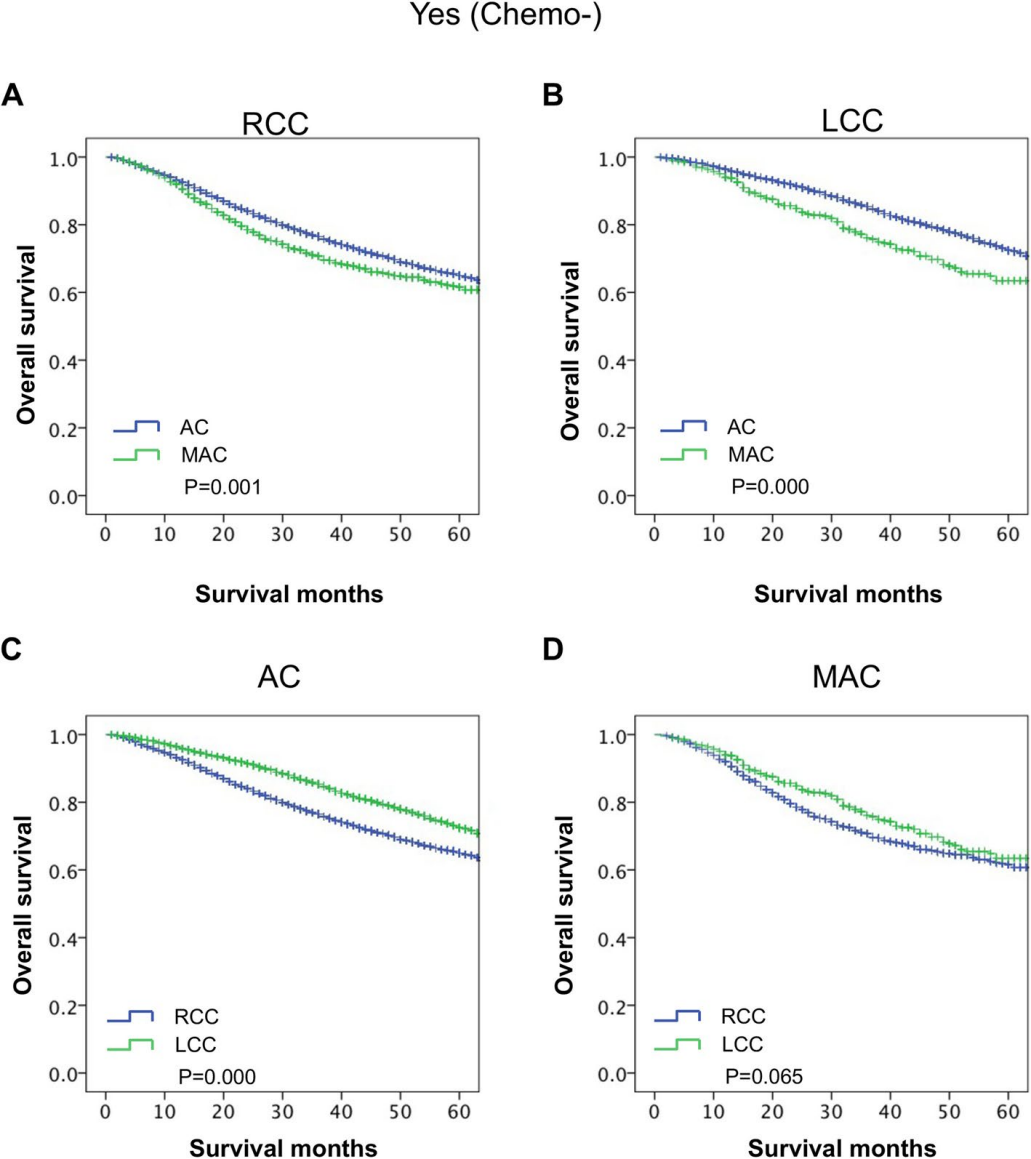
The impact of the interplay of PTL and histological subtype on long-term survival of stage III CC patients with chemotherapy. **A** The stratified analysis survival curves showed that AC had a better OS than MAC in the RCC group. **B** The stratified analysis survival curves showed that AC had a better OS than MAC in the LCC group. **C** The stratified analysis survival curves showed that LCC had a better OS than RCC in the AC group. **D** The stratified analysis survival curves showed that LCC had comparable OS with RCC in the MAC group

First, we analyzed the survival differences between MAC and AC stratified by PTL. The results showed that the OS of the MAC group was poorer than that of the AC group in the RCC subgroup (*P* ≤ *0.001*, Fig. 4A). Additionally, the OS of the MAC group was also poorer than that of the AC group in the LCC subgroup (*P* ≤ 0.001, Fig. 4B). In addition, the survival differences between RCC and LCC were also stratified by histological subtype. The results showed that the OS of the RCC group was poorer than that of the LCC group in the AC subgroup (*P* ≤ *0.001*, Fig. 4C), but the OS of the RCC group was comparable to that of the LCC group in the MAC subgroup (*P* = 0.065, Fig. 4D). These results indicated that MAC was a poor prognostic factor for the OS of stage III CC patients with chemotherapy regardless of PTL, but PTL was not a prognostic factor for stage III MAC patients with chemotherapy.

### The long-term survival difference among the subgroups of RCC/MAC, RCC/AC, LCC/MAC and LCC/AC.

**Fig. 5:**
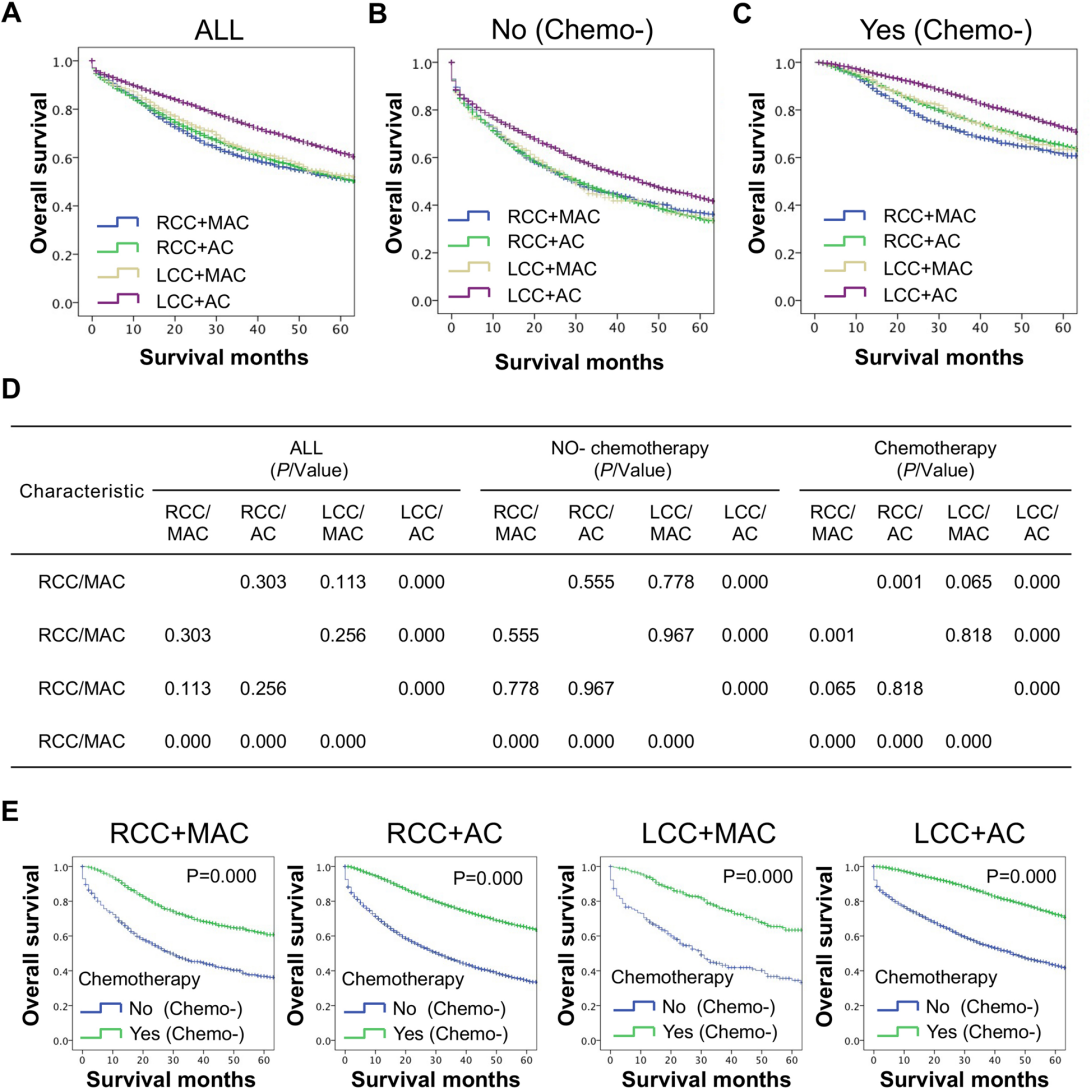
The long-term survival difference between the four subgroups, RCC/MAC, RCC/AC, LCC/MAC and LCC/AC. **A** The survival curves showed that LCC/AC had the best OS compared with the other three subgroups, which had similar OS. **B** For patients without chemotherapy, LCC/AC had the best OS compared with the other three subgroups, which had similar OS. **C** For patients with chemotherapy, LCC/AC had the best OS, followed by RCC/AC and LCC/MAC, which had similar OS, and RCC/MAC, which had the worst OS. **D** The results showed statistical analysis for Figures A, B and C. **E** The results showed that patients with chemotherapy had a better OS than those without chemotherapy in the RCC/MAC, RCC/AC, LCC/MAC, and LCC/AC groups

We classified stage III CC patients into four groups based on PTL and histological subtype: RCC/MAC, RCC/AC, LCC/MAC and LCC/AC. The distribution of chemotherapy among them is shown in Table 5. We found that the LCC/AC group had the best OS compared with the other three subgroups, while the OS of the RCC/MAC, RCC/AC and LCC/MAC groups were similar to each other (Fig. 5A and 5D). In addition, the survival differences among RCC/MAC, RCC/AC, LCC/MAC and LCC/AC stratified by chemotherapy were also analyzed (Fig. 5B-D). In the nonchemotherapy hierarchy, LCC/AC had the best OS compared with the other three subgroups (*P* ≤ *0.001*, Fig. 5B and 5D). In the chemotherapy hierarchy, LCC/AC also had the best OS (*P* ≤ 0.001, Fig. 5C and 5D), but RCC/MAC had the worst OS (*P* ≤ *0.001*, Fig. 5C and 5D).

**Table 5:** The distribution of chemotherapy among the four subgroups after combining PTL and histology subtype

	Total (28,765)	Chemotherapy		*P*/Value
		No/Unknown (11,645)	Yes (17,120)	
Histology/PTL				≤ 0.001
RCC/MAC	2327(8.1)	949(8.1)	1378(8.0)	
RCC/AC	15,103(52.5)	6583(56.5)	8520(49.8)	
LCC/MAC	752(2.6)	284(2.4)	468(2.7)	
LCC/AC	10,583(36.8)	3829(32.9)	6754(39.5)

To further explore the impact of chemotherapy on the survival of the four groups of RCC/MAC, RCC/AC, LCC/MAC and LCC/AC, potential survival differences among them were analyzed. The results showed that patients with chemotherapy had a better OS than those without chemotherapy in the RCC/MAC, RCC/AC, LCC/MAC and LCC/AC subgroups (all *P* ≤ *0.001*, Fig. 5E).

### The impact of the interplay of PTL and histological subtype on the survival of stage III CC patients without chemotherapy.

**Fig. 6:**
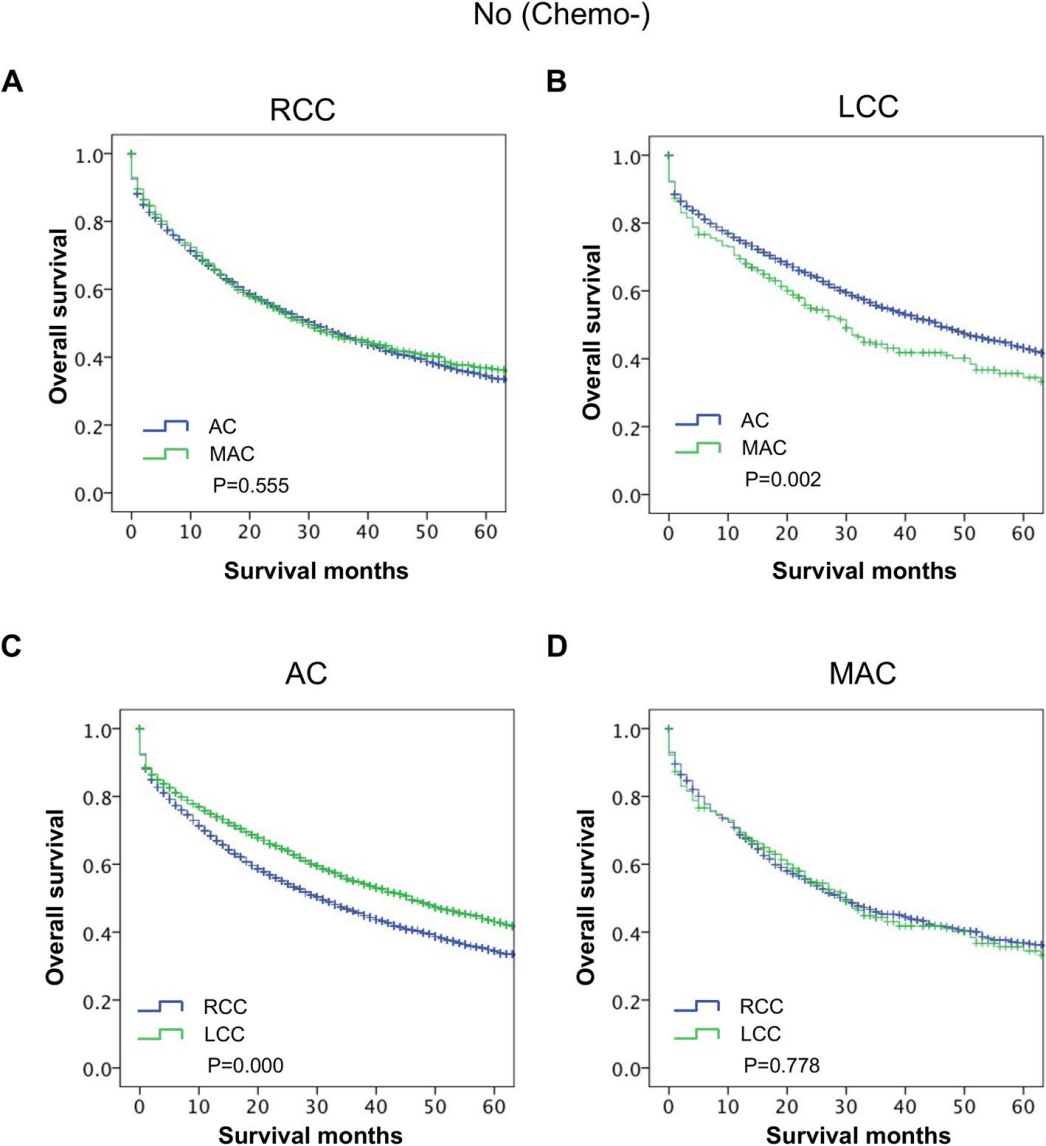
The impact of the interplay of PTL and histological subtype on the long-term survival of stage III CC patients without chemotherapy. **A** The stratified analysis survival curves showed that AC had comparable OS with MAC in the RCC group. **B** The stratified analysis survival curves showed that AC had a better OS than MAC in the LCC group. **C** The stratified analysis survival curves showed that LCC had a better OS than RCC in the AC group. **D** The stratified analysis survival curves showed that LCC had comparable OS to RCC in the MAC group

To further explore the impact of the interplay of PTL and histological subtype on the OS of stage III CC patients, we analyzed the survival differences via stratification analyses in the nonchemotherapy group. First, the survival differences between MAC and AC were stratified by PTL. The results showed that the OS of the MAC group was similar to that of the AC group in the RCC subgroup (*P* = 0.555, Fig. 6A). However, the OS of the MAC group was poorer than that of the AC group in the LCC subgroup (*P* = 0.002, Fig. 6B). Second, the survival difference analyses between RCC and LCC were stratified by histological subtype. The results showed that the OS of RCC was poorer than that of LCC in the AC subgroup (*P* ≤ *0.001*, Fig. 6C), but the OS of RCC was comparable to that of LCC in the MAC subgroup (*P* = 0.778, Fig. 6D). These results indicated that in stage III CC patients without chemotherapy, MAC is a poor indicator of OS for LCC but not for RCC. RCC is a poor indicator of OS in the AC but not in the MAC.

### Risk factors for long-term survival of stage III CC patients

**Fig. 7:**
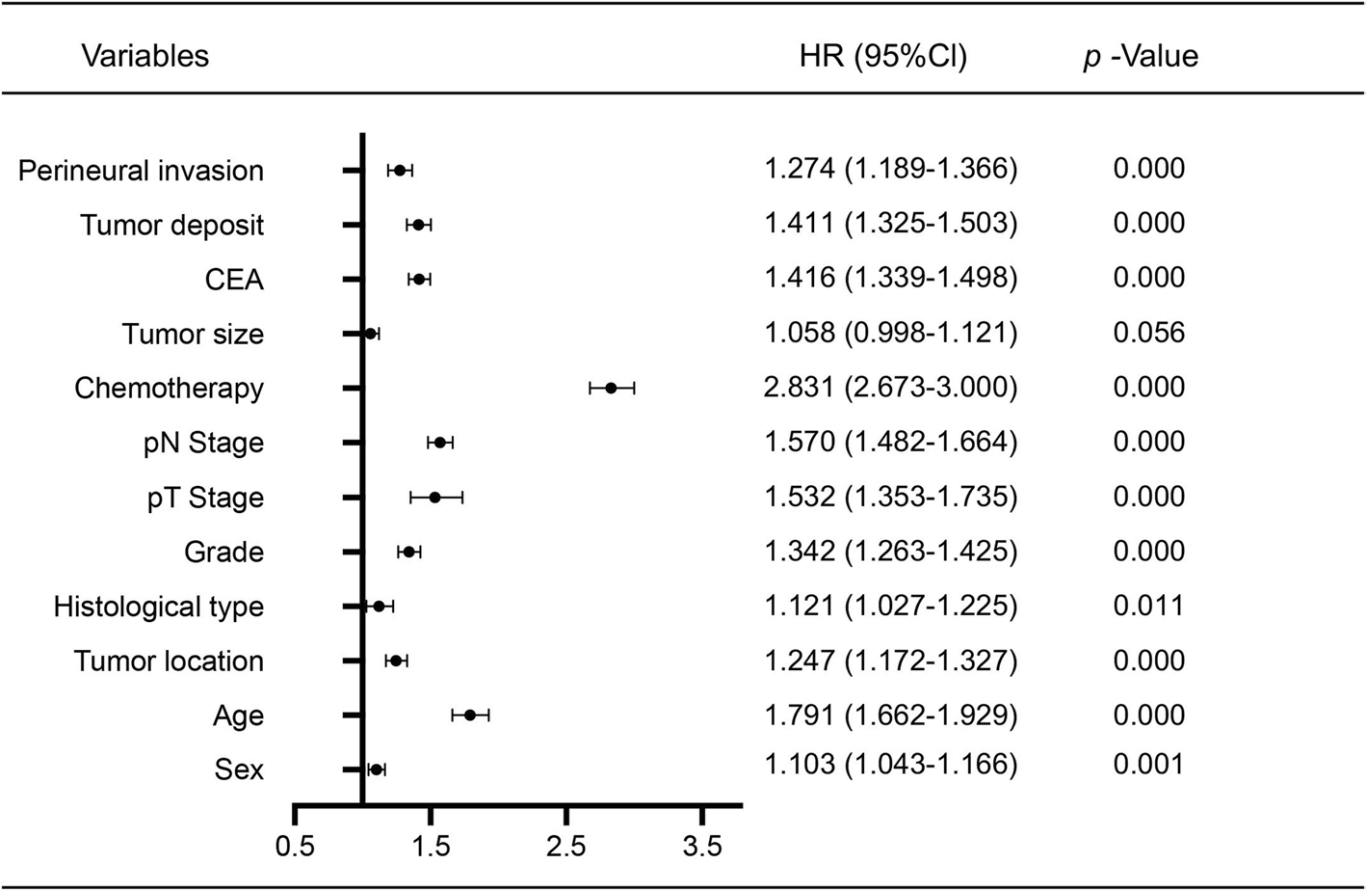
Risk factors for long-term survival of stage III CC patients. Multivariate analysis of factors associated with OS in stage III CC patients

The risk factors for survival of stage III CC patients were explored via multivariable analyses (Fig. 7). Multivariate analysis revealed that OS was significantly dependent on sex, age, PTL, histological subtype, grade, p–T stage, p-N stage, chemotherapy, CEA, tumor deposits and perineural invasion in stage III CC patients (*P* < 0.05). However, tumor size was not significantly associated with OS in stage III CC patients in multivariable analyses (*P* > 0.05). Based on the results, sex, age, PTL, histological subtype, grade, p–T stage, p-N stage, chemotherapy, CEA, tumor deposits and perineural invasion were significant independent prognostic factors for OS in stage III CC patients.

## Discussion

Stage III CC patients account for a considerable proportion of cases: it has been reported that more than one-third of CC patients are stage III patients [23, 24]. In patients with stage III CC, chemotherapy is recommended and could reduce the risk of mortality and recurrence [25, 26]. Survival benefits from chemotherapy for patients with stage III CC are generally accepted, and many studies have shown that PTL is associated with long-term survival in stage III CC patients [27–29]. However, the differences in MAC and AC with respect to the prognosis of CC patients remains unclear [30–32]. Interestingly, the survival benefit from chemotherapy is greater for patients with LCC than for those with RCC [33, 34]. In addition, MAC has also been observed more often in RCC than in LCC [13, 35, 36]. Therefore, it is meaningful to identify the separate and joint effects of chemotherapy, PTL and histological subtype on the survival of patients with stage III CC.

Chemotherapy and LCC were associated with favorable OS in patients with stage III CC in this study. Importantly, we found that the OS of MAC was poorer than that of AC in stage III CC. MAC accounted for 10.7% of all CC cases and was more frequently found in RCC (RCC: 75.6% vs. LCC: 24.4%). The survival was different between PTL or histological subtype via stratification of chemotherapy. These results indicated that PTL might be an independent prognostic factor affecting the OS of patients with stage III CC regardless of chemotherapy, and histological subtype might be an independent prognostic factor affecting the OS of stage III CC patients with chemotherapy, which indicated that the prognosis of stage III CC was strongly associated with PTL and histological subtype. One novel aspect of this study is the interplay analyses of PTL and histological subtype on survival of stage III CC patients. In the LCC subgroup, MAC had worse OS than AC regardless of chemotherapy. In the RCC subgroup, MAC also had worse OS than AC in patients with chemotherapy, but MAC had similar OS to AC in patients without chemotherapy, which might be explained by the survival disadvantage of MAC. Additionally, it also indicated that the survival advantage of chemotherapy for AC is greater than that for MAC, which meant that the impact of chemotherapy on survival is greater than that of histological subtype. Surprisingly, in the AC subgroup, RCC had worse OS than LCC regardless of chemotherapy. In the MAC cohort, RCC had comparable OS to LCC regardless of chemotherapy. The reason for this finding might be that the MAC histological subtype offset the survival advantage brought by LCC, which indicated that the impact of histological subtype on survival is close to the impact of PTL on survival.

Furthermore, after combining analyses of PTL and histological subtype, the results showed that the LCC/AC subgroup exhibited better OS than the other three subgroups in the whole cohort, while the OS of the RCC/MAC, RCC/AC and LCC/MAC groups were similar to each other. When these four subgroups were stratified by chemotherapy, the results in the nonchemotherapy cohort showed the same results as the whole cohort, while the OS of the RCC/MAC group was different from that of the RCC/AC and LCC/MAC groups and became the worst in the chemotherapy cohort, which could also obtain a survival benefit from chemotherapy. This indicated that chemotherapy is still needed for RCC/MAC.

There are also several limitations to the present study. First, this is a retrospective study of public databases, which has potential selection bias. Second, due to limitations of the SEER database, information on the type of chemotherapy regimen, adherence and completion rates were not available. Third, there was a lack of support from multicenter perspective randomized controlled trials.

## Conclusion

In conclusion, this study found survival differences in the subgroups of stage III CC, and chemotherapy, LCC or AC was associated with improved OS. LCC/AC had the best OS, while RCC/MAC had the worst OS but could also benefit from chemotherapy. The impact of chemotherapy on survival is greater than that of histological subtype. However, the impact of histological subtype on survival is close to that of PTL.

## Data Availability

The datasets used and/or analyzed during the current study are available from the corresponding author on reasonable request.

## Abbreviations

CC: Colon cancer
PTL: Primary tumor location
MAC: Mucinous adenocarcinoma
AC: Adenocarcinoma
OS: Overall Survival
SEER: Surveillance, Epidemiology, and End Results
LCC: Left-sided Colon Cancer
RCC: Right-sided Colon Cancer

## References

[1] Siegel RL, Miller KD, Goding Sauer A, Fedewa SA, Butterly LF, Anderson JC, Cercek A, Smith RA, Jemal A (2020). Colorectal cancer statistics, 2020. CA Cancer J Clin.

[2] Siegel RL, Miller KD, Jemal A (2020). Cancer statistics, 2020. CA Cancer J Clin.

[3] NIH consensus conference (1990). Adjuvant therapy for patients with colon and rectal cancer. JAMA.

[4] Benson AB, 3rd, Venook AP, Bekaii-Saab T, Chan E, Chen YJ, Cooper HS, Engstrom PF, Enzinger PC, Fenton MJ, Fuchs CS, et al. Colon cancer, version 3.2014. J Natl Compr Canc Netw. 2014;12(7):1028–59. 10.6004/jnccn.2014.0099 24994923

[5] Guinney J, Dienstmann R, Wang X, de Reyniès A, Schlicker A, Soneson C, Marisa L, Roepman P, Nyamundanda G, Angelino P (2015). The consensus molecular subtypes of colorectal cancer. Nat Med.

[6] Missiaglia E, Jacobs B, D'Ario G, Di Narzo AF, Soneson C, Budinska E, Popovici V, Vecchione L, Gerster S, Yan P (2014). Distal and proximal colon cancers differ in terms of molecular, pathological, and clinical features. Ann Oncol.

[7] André T, Boni C, Navarro M, Tabernero J, Hickish T, Topham C, Bonetti A, Clingan P, Bridgewater J, Rivera F (2009). Improved overall survival with oxaliplatin, fluorouracil, and leucovorin as adjuvant treatment in stage II or III colon cancer in the MOSAIC trial. J Clin Oncol.

[8] You XH, Jiang YH, Fang Z, Sun F, Li Y, Wang W, Xia ZJ, Wang XZ, Ying HQ: Chemotherapy plus bevacizumab as an optimal first-line therapeutic treatment for patients with right-sided metastatic colon cancer: a meta-analysis of first-line clinical trials. *ESMO Open* 2020, 4(Suppl 2). 10.1136/esmoopen-2019-000605 PMC7064070 32132090

[9] Kim ST, Lee SJ, Lee J, Park SH, Park JO, Lim HY, Kang WK, Park YS (2017). The Impact of Microsatellite Instability Status and Sidedness of the Primary Tumor on the Effect of Cetuximab-Containing Chemotherapy in Patients with Metastatic Colorectal Cancer. J Cancer.

[10] Taieb J, Kourie HR, Emile JF, Le Malicot K, Balogoun R, Tabernero J, Mini E, Folprecht G, Van Laethem JL, Mulot C (2018). Association of Prognostic Value of Primary Tumor Location in Stage III Colon Cancer With RAS and BRAF Mutational Status. JAMA Oncol.

[11] Huang J, Huang Q, Tang R, Chen G, Zhang Y, He R, Zu X, Fu K, Peng X, Xiao S (2020). Hemicolectomy Does Not Provide Survival Benefit for Right-Sided Mucinous Colon Adenocarcinoma. Front Oncol.

[12] Hugen N, Brown G, Glynne-Jones R, de Wilt JH, Nagtegaal ID (2016). Advances in the care of patients with mucinous colorectal cancer. Nat Rev Clin Oncol.

[13] Luo C, Cen S, Ding G, Wu W (2019). Mucinous colorectal adenocarcinoma: clinical pathology and treatment options. Cancer Commun (Lond).

[14] Huang J, Chen G, Liu H, Zhang Y, Tang R, Huang Q, Fu K, Peng X, Xiao S (2020). Surgery improves the prognosis of colon mucinous adenocarcinoma with liver metastases: a SEER-based study. BMC Cancer.

[15] Nozoe T, Anai H, Nasu S, Sugimachi K (2000). Clinicopathological characteristics of mucinous carcinoma of the colon and rectum. J Surg Oncol.

[16] Yu D, Gao P, Song Y, Yang Y, Chen X, Sun Y, Li A, Wang Z (2018). The differences on efficacy of oxaliplatin in locally advanced colon cancer between mucinous and nonmucinous adenocarcinoma. Cancer Med.

[17] Graziano F, Ruzzo A, Giacomini E, Ricciardi T, Aprile G, Loupakis F, Lorenzini P, Ongaro E, Zoratto F, Catalano V (2017). Glycolysis gene expression analysis and selective metabolic advantage in the clinical progression of colorectal cancer. Pharmacogenomics J.

[18] Luo C, Cen S, Ying J, Wang X, Fu Z, Liu P, Wu W, Ding G (2019). Tumor clinicopathological characteristics and their prognostic value in mucinous colorectal carcinoma. Future Oncol.

[19] Zhou YW, Long YX, Chen Y, Liu JY, Pu D, Huang JY, Bi F, Li Q, Gou HF, Qiu M (2021). First-line therapy of bevacizumab plus chemotherapy versus cetuximab plus chemotherapy for metastatic colorectal cancer patients with mucinous adenocarcinoma or mucinous component. Cancer Med.

[20] Lan YT, Chang SC, Lin PC, Lin CC, Lin HH, Huang SC, Lin CH, Liang WY, Chen WS, Jiang JK (2021). Clinicopathological and Molecular Features of Colorectal Cancer Patients With Mucinous and Non-Mucinous Adenocarcinoma. Front Oncol.

[21] Zenger S, Gurbuz B, Can U, Balik E, Bugra D (2020). Clinicopathologic features and prognosis of histologic subtypes in the right-sided colon cancer. J buon.

[22] Zhou C, Zhang Y, Hu X, Fang M, Xiao S (2021). The effect of marital and insurance status on the survival of elderly patients with stage M1b colon cancer: a SEER-based study. BMC Cancer.

[23] Chagpar R, Xing Y, Chiang YJ, Feig BW, Chang GJ, You YN, Cormier JN (2012). Adherence to stage-specific treatment guidelines for patients with colon cancer. J Clin Oncol.

[24] Schmoll HJ, Tabernero J, Maroun J, de Braud F, Price T, Van Cutsem E, Hill M, Hoersch S, Rittweger K, Haller DG (2015). Capecitabine Plus Oxaliplatin Compared With Fluorouracil/Folinic Acid As Adjuvant Therapy for Stage III Colon Cancer: Final Results of the NO16968 Randomized Controlled Phase III Trial. J Clin Oncol.

[25] Taieb J, Gallois C: Adjuvant Chemotherapy for Stage III Colon Cancer. *Cancers (Basel)* 2020, 12(9). 10.3390/cancers12092679 PMC7564362 32961795

[26] Mlecnik B, Bifulco C, Bindea G, Marliot F, Lugli A, Lee JJ, Zlobec I, Rau TT, Berger MD, Nagtegaal ID (2020). Multicenter International Society for Immunotherapy of Cancer Study of the Consensus Immunoscore for the Prediction of Survival and Response to Chemotherapy in Stage III Colon Cancer. J Clin Oncol.

[27] Aggarwal H, Sheffield KM, Li L, Lenis D, Sorg R, Barzi A, Miksad R (2020). Primary tumor location and survival in colorectal cancer: A retrospective cohort study. World J Gastrointest Oncol.

[28] Cascinu S, Poli D, Zaniboni A, Lonardi S, Labianca R, Sobrero A, Rosati G, Di Bartolomeo M, Scartozzi M, Zagonel V et al. The prognostic impact of primary tumour location in patients with stage II and stage III colon cancer receiving adjuvant therapy. A GISCAD analysis from three large randomised trials. Eur J Cancer. 2019;111:1–7. 10.1016/j.ejca.2019.01.020 30797014

[29] Taieb J, Shi Q, Pederson L, Alberts S, Wolmark N, Van Cutsem E, de Gramont A, Kerr R, Grothey A, Lonardi S (2019). Prognosis of microsatellite instability and/or mismatch repair deficiency stage III colon cancer patients after disease recurrence following adjuvant treatment: results of an ACCENT pooled analysis of seven studies. Ann Oncol.

[30] Verhulst J, Ferdinande L, Demetter P, Ceelen W (2012). Mucinous subtype as prognostic factor in colorectal cancer: a systematic review and meta-analysis. J Clin Pathol.

[31] Park JS, Huh JW, Park YA, Cho YB, Yun SH, Kim HC, Lee WY, Chun HK (2015). Prognostic comparison between mucinous and nonmucinous adenocarcinoma in colorectal cancer. Medicine (Baltimore).

[32] Hogan J, Burke JP, Samaha G, Condon E, Waldron D, Faul P, Coffey JC (2014). Overall survival is improved in mucinous adenocarcinoma of the colon. Int J Colorectal Dis.

[33] Stintzing S, Tejpar S, Gibbs P, Thiebach L, Lenz HJ (2017). Understanding the role of primary tumour localisation in colorectal cancer treatment and outcomes. Eur J Cancer.

[34] Shen H, Yang J, Huang Q, Jiang MJ, Tan YN, Fu JF, Zhu LZ, Fang XF, Yuan Y (2015). Different treatment strategies and molecular features between right-sided and left-sided colon cancers. World J Gastroenterol.

[35] Chen J, Zhou L, Gao J, Lu T, Wang J, Wu H, Liang Z (2020). Clinicopathological Characteristics and Mutation Spectrum of Colorectal Adenocarcinoma With Mucinous Component in a Chinese Cohort: Comparison With Classical Adenocarcinoma. Front Oncol.

[36] Zheng C, Jiang F, Lin H, Li S (2019). Clinical characteristics and prognosis of different primary tumor location in colorectal cancer: a population-based cohort study. Clin Transl Oncol.

